# Clozapine induced myocarditis: a case report

**DOI:** 10.1192/j.eurpsy.2022.1888

**Published:** 2022-09-01

**Authors:** M. López Isern, D. Paiva Pajares, A. Martínez Muelas, A. Arévalo Sánchez, M. Sánchez Pérez

**Affiliations:** Hospital Sagrat Cor, Hermanas Hospitalarias, Psychiatry, Martorell (Barcelona), Spain

**Keywords:** clozapine, schizoaffectivedisorder, Psychofarmacology, myocarditis

## Abstract

**Introduction:**

Clozapine is one of the most effective antipsychotic drugs. On the other hand, it can cause serious side effects which have to be monitored. An adverse effect is myocarditis, a type B Clozapine reaction that can be fatal if it is not early diagnosed.

**Objectives:**

To report a case of a patient with Clozapine induced myocarditis.

**Methods:**

A 48 years old women with a schizoaffective disorder was admitted to our Hospital due to a clinical decompensation. She had a manic episode with psychotic symptoms (persecutory delusions and auditive hallucinations). Clozapine was introduced after there were no improvement with Olanzapine, Risperidone and Valproic Acid. A dose increase was made reaching 100 mg/day the first week and 200 mg/day the second week. The third week she started with a 39ºC fever, decreased oxygen saturation, leukocytosis (9560 10^3^/mm^3^), elevated PCR (210 mg/l) and elevated troponins (52,88 ng/l). EKG and other medical tests did not show alterations. There was not found a clear etiology, so Clozapine was retired as a cautionary measure. The differential diagnosis for etiology included viral infections, Clozapine induced myocarditis or idiopathic.

**Results:**

A few days after the withdrawal of Clozapine, cardiac symptoms improved, suggesting it was the most probable etiology.

**Conclusions:**

Although it is not very likely to occur, it is important to consider myocarditis as a sever Clozapine side effect.

**Disclosure:**

No significant relationships.

